# ‘Doing hymen reconstruction’: an analysis of perceptions and experiences of Flemish gynaecologists

**DOI:** 10.1186/s12905-018-0587-z

**Published:** 2018-06-13

**Authors:** Els Leye, Emilomo Ogbe, Maaike Heyerick

**Affiliations:** 1 International Centre for Reproductive Health, Faculty of Medicine, Ghent University, C. Heymanslaan 10, entrance 75, UZP 114, 9000 Ghent, Belgium; 2 Faculty of Medicine, Ghent University, Ghent, Belgium

**Keywords:** Hymen reconstruction, Belgium, Gynaecologists, Survey

## Abstract

**Background:**

Hymen reconstruction (HR) involves the restoration of the hymeneal membrane’s gross anatomical integrity. Among the medical profession, hymen reconstruction receives particular attention and its necessity is debated because the surgery is not medically indicated, and often reveals conflicting social norms on virginity and marriageability between health professionals and their patients. The focus of this paper is not to address the many open questions that the ethics and politics around HR reveal, but rather aims at contributing to the much-needed empirical evidence. It presents findings of a study conducted in Belgium (Flanders region), among gynaecologists that aimed at assessing their knowledge, views, and experiences on hymen reconstruction.

**Methods:**

A digital self-administered questionnaire-based survey was sent to Flemish gynaecologists and trainees in Flanders registered with the Flemish Society of Obstetrics and Gynaecology (VVOG).

**Results:**

Hundred-and-nine questionnaires were completed. The majority of the respondents (73%) had requests to perform HR. Knowledge and technical skills about HR were considered to be sufficient (69%), even though HR does not seem to be integrated in medical curricula or post-graduate training. Most respondents (72%) would favour the publication of a guideline by their professional organisation. Few respondents discuss alternative options with the patient (19%) and half of the respondents reject to perform HR (49%). The majority of our respondents are against reimbursement of the surgery (70%). Not even half of our respondents believes that a patient is at risk of further violence (47%). 7% of the respondents mentioned complications, but the majority was able to perform a follow up consultation.

**Conclusions:**

The responses of this survey cannot be generalised to the entire population of gynaecologists in Flanders, but do provide insights in how gynaecologists confronted with HR are approaching such requests, and thus contributes to the empirical evidence. Our paper showed that many Flemish gynaecologist are likely to encounter requests for hymenoplasty, but that a majority would not perform the surgery. There seems to be a lack of guidance and debate in Flanders on the social and moral dimensions of HR, and a number of complexities were revealed when gynaecologists address HR that need further research.

**Electronic supplementary material:**

The online version of this article (10.1186/s12905-018-0587-z) contains supplementary material, which is available to authorized users.

## Background

Hymen reconstruction (HR), which is also referred to as hymenorrhaphy or hymenoplasty, is defined within the context of this paper as the process of restoring the hymeneal membrane’s gross anatomical integrity [1]. HR is framed both as an operation that promotes certain harmful gender norms and at the same time, serves as a way of protecting young women and girls from the stigma or violence associated with ‘losing their virginity before marriage’ [2, 3]. Some studies showed that for some women who undergo hymen reconstruction, it is seen as a pragmatic choice, that allows them to meet societal expectations of virginity, marriageability and also avoid further acts of violence [4–7]. Among the medical profession, hymen reconstruction receives particular attention and is debated because the surgery is not medically indicated and reveals contradictory social norms regarding virginity and marriageability for girls/women [8, 9]. The belief that bed sheets must be stained with blood after first intercourse during the wedding night, as a sign of virginity, contradicts with the medical evidence that blood loss after first coitus does not always occur [10–12], and with the fact that there is a wide variety of appearances of the hymen in virgin girls and women [13, 14]. Moreover, an ‘intact hymen’ is not always a sign of not having engaged in sexual activity as there are other ways of preserving virginity while at the same time having engaged in sexual intercourse [15].

Some studies among health professionals provide insights in the varying views and opinions of these professionals on virginity and HR, and how this affects the provision of care for women requesting HR. Loeber for example, discusses the difference in discourses and understandings of virginity among western and non-western societies and how this might explain the different attitudes of health care practitioners towards hymen reconstruction surgery in the Netherlands [6]. Another study in the Netherlands, by Ayuandini, shows how doctors perceive the request for HR as a cultural problem and not a medical/anatomical issue [16]. Juth discusses how perceptions of requests of HR are influenced by value systems and beliefs of attending physicians in Sweden; showing that physicians who were willing to help women requesting these procedures and had performed these procedures before were more likely to perceive HR ‘as not so bad’ compared to their colleagues who did not [17]. In Tehran, a study done among physicians revealed that though performing HR has ‘punitive consequences’ by law (doctors could have their license suspended if they performed the surgery), most doctors still felt a moral obligation to perform the procedure, because of the possibilities of women experiencing violence, if they are perceived to not be virgins when they get married. The responding physicians also did not have any ethical objections to performing the surgery [18]. In Sweden, a qualitative study done among midwives, who were mostly from ‘western countries’, framed HR as a ‘misogynistic practice’ and respondents felt there was a need to provide ‘culturally sensitive’ care to women who sought HR [19]. In Belgium, the number of HR is unknown and no studies on perceptions of health professionals have been conducted so far, nor on the views of women requesting such surgery. HR are reimbursed in Belgium by the social security, and are classified under the nomenclatura “vaginoplasty” or “vulvaplasty”; but no specific category is available for HR. This makes it difficult to adequately estimate and monitor the number of reconstructions done in Belgium, although Amy mentioned an increasing number of women requesting a hymen reconstruction procedure or ‘certificates of virginity’ from health care professionals (HCP) [20]. At regular intervals, proposals for resolutions are introduced by politicians aiming at abolishing the reimbursements by the social security, whereby it is argued that the principle of virginity is in contradiction with the basic principle of democracy, and that reimbursing a surgery that is not medically indicated is not justifiable [21].

The only data that are available are from the French speaking part of Belgium (Wallonia). A survey from 2007 by the Groupement de Gynécologues Obstétriciens de Langue Française de Belgique (GGOLFB) (*n* = 254), revealed that there were 310 applications for a virginity certificate and 238 hymen reconstructions performed, that year [22]. In Flanders, 3046 surgeries were classified as vagino and vulva-plasty in 2015. The majority concerns labia corrections and reconstructions after female genital mutilation/cutting; the share of HR is unknown (personal communication RIZIV to first author, August 2017).

The focus of this paper is not to address the many open questions that the ethics and politics around HR reveal, but rather aims at contributing to the much-needed empirical evidence. Indeed, despite some studies have been published in recent years, there is still a scarcity of empirical evidence on needs and motivations of women requesting and health professionals providing HR [4]. This paper presents findings of a study conducted in Belgium (Flanders region), among gynaecologists that aimed at assessing their knowledge, views, and experiences on hymen reconstruction.

## Methods

This study on hymen reconstruction among HCP in Flanders was done through a web based survey, using a semi-structured questionnaire (Additional file 1). This instrument was developed after a systematic literature review revealing issues, gaps and discourses around the procedure in Europe and Belgium, and after attending a HR surgery in Ghent University Hospital. The questionnaire was pre-tested among some gynaecologists, after which it was made available on the website of the Vlaamse Vereniging Vereniging voor Obstetrie en Gynecologie vzw (Flemish Society of Obstetrics and Gynaecology (VVOG). A mailing was done in June 2011 to all members of the VVOG in which participation in the survey was requested, and a second mailing in November 2011. At the time of the survey, there were slightly over 500 registered gynaecologists and assistant-gynaecologists trainees in Flanders and 98% are members of this Society [23]. At the closure of the study, 109 questionnaires had been filled out, providing a response rate of 22%. The study was carried out between May 2011 and February 2012, using convenience sampling.

The Critical Appraisal Skills Programme (CASP) was used to assess the survey’s accuracy, credibility and relevance [24], whereby maximal correspondence to the CASP criteria on research design, sampling, data collection, reflexivity, ethical issues, data analysis, conclusions and value of the research was sought.

The questionnaire included 40 questions collecting information and asking for personal comments on:

1. socio-demographics of respondents (age, gender, type of practice (private or public), location of practice (city or rural) and number of requests of HR in past 12 months);
2. training and guidelines (if HR was included in basic and postgraduate training), and knowledge about existing guidelines on HR (from Belgian or international organisation);
3. need for more information/knowledge (should HR be included in curricula; more information during training would have been welcome; preference to have a guideline from VVOG);
4. information collected during intake (among others, reasons why the patient requests HR);
5. alternatives for HR suggested to patients;
6. if the respondent is performing HR or not, and why (not), whether there were complications, whether they do follow-up and why (not);
7. whether they agree with the reimbursement of HR by the social security; and
8. their personal opinions on a number of statements regarding HR.

Confidentiality was assured to all respondents. Data was analysed using SPSS 19 for Windows. Ethical clearance was obtained from the ethical committee of the Ghent University Hospital.

## Results

### Characteristics of the respondents

Of the 104 respondents that answered questions related to age, gender and place of work, thirty seven (35.5%) were 30–40 years old, 39 (37.5%) were 41–50 years old and 28 (27%) were older than 51. Forty-one (39%) respondents were male and 63 (61%) were female. Forty-five (43%) respondents worked in a public hospital, five in a private hospital (5%), 21 (20%) in a university hospital and 33 (32%) in a combination of these.

### Knowledge of the respondents

#### Knowledge of hymen reconstruction

Of 109 respondents, only 12 respondents (11%) had heard about hymen reconstruction during basic medical training and 37 during postgraduate training (34%). Forty respondents (37%) indicated that additional information in the medical curricula about hymen reconstruction would be desirable, while the majority thought this was redundant.

#### Knowledge of existing guidelines

Eighty-eight respondents (81%) had no knowledge of guidelines in Belgium or abroad. Respondents who were aware of a guideline, mentioned guidelines from the UK, and the Netherlands, as well as the position statement of the French College of Obstetricians and Gynaecologists. Seventy-nine respondents (72%) would welcome a guideline by the Flemish Society of Obstetrics and Gynaecology.

#### Personal comments

The questionnaire allowed for the provision of personal comments and opinions. With regard to the need for more information, two respondents indicated that more information on social and cultural aspects of hymen reconstructions, and on the current debate regarding hymen repair, would be welcome. These respondents also indicated that no further information is required on the technical aspects of the surgery. With regard to the need for guidelines, two respondents thought that a position statement of the VVOG could provide more clarity, while two others thought this was not necessary as it is too patronising and the decision ultimately lies with the individual gynaecologist. A respondent suggested that opinion papers by supporters and opponents of the procedure could equally be helpful.

### Views/opinions of the respondents

Seventy-five respondents (69%) felt they had sufficient knowledge and technical skills to perform the operation, while 20 respondents (18%) thought they lacked the knowledge and skills to perform this type of surgery. Eighty respondents (73%) thought the surgery was medically unnecessary while only eight respondents considered it a medical necessity.

#### Autonomy of the patient

Sixty-three out of 105 respondents (60%) agreed to the statement that the patient has the right to decide over her body when it comes to deciding to have HR, irrespective of the personal principles of the HCP. Twenty respondents (19%) did not agree with this statement and 22 (21%) were neutral. Respondents had varying opinions about the statement that ‘the surgery violates the right to physical integrity and self-determination of the patient’. An equal number of respondents (34, 31%) ‘agreed’ and ‘did not agree’ with this statement, and 37 respondents (34%) picked ‘did not know’, as a response.

#### Perception of harm to the patient

Forty-nine of the 105 (47%) respondents acknowledged that the patient was in danger of becoming a victim of violence if her hymen would not be restored, while 19 respondents (18%) did not agree with this and 37 had no opinion (35%).

A total of 37 respondents (35%) out of 105 did not agree with the statement that hymen reconstruction is a harmless and reversible type of surgery. Twenty-nine respondents were neutral (28%), while 39 agreed (37%).

When asked if hymen reconstructions maintain a double standard (virginity is required for a female, not for a male), a total of 69 respondents agreed (66%). Moreover, 75 respondents (71%) thought that the surgery maintains the virginity myth.

Only 24 of the 105 respondents agreed with the statement that hymen repair can deconstruct the double standard (23%), while 46 respondents (44%) had no opinion and 35, disagreed (33%).

#### Reimbursement of the surgery by the social security system

A majority of the respondents, 85, which is more than 70 % of the respondents, did not think that the surgery should be reimbursed by the social security scheme. The main argument against reimbursement is the fact that the procedure has no medical indication. Those in favour of reimbursement argued that it contributes to the psychological health, referring to the fact that other medically ‘unnecessary operations’ are equally reimbursed and it prevents the patient from looking for cheaper, more dangerous options.

### Experiences of the respondents

#### Demand for hymen reconstruction

Eighty respondents (73%) received a request to perform HR during their career; while in the past 12 months 21 (26,3%) did not receive any request, 19 respondents (24%) received one to two requests and 20 respondents (25%) received three or more requests. Twenty-nine respondents (27%) stated that they did not receive any request for a HR in that period.

#### Assessment of patients

The respondents assessed the patients’ background during the consultation. Of the 80 respondents who answered this section of the survey, questions were asked most commonly on the patients’ motivation for requesting the hymen reconstruction procedure (70 respondents, 88%), age (70%, 56 respondents), relational status (69%, 55 respondents), religion (61%, 48 respondents), ethnicity (59%, 47 respondents) and the existence of a close friend or confidante (28%, 22 respondents). Forty-three respondents (45%) also assessed the patient’s knowledge of the female genital anatomy and the role of the hymen.

Of the 80 respondents who received a request to perform hymen reconstructions, the majority - 70 respondents (88%) - asked for the reason behind the request. Thirty-two respondents (59%) provided more information on why they probed for this. Three main reasons were put forward: 1) the need for more clarity on a patient’s reason for the request in order to assess the fear for retribution (59%); 2) to sensitise the patient about the controversies surrounding hymen repairs and 3) purely out of interest. Seven respondents (22%) said posing the question was not necessary. The respondents shared insights in the reasons put forward by patients to request a repair. These included cultural norms, pressure by the family or the future family in law, religious reasons, fear of violence, respect and affection for the future husband or family. Aesthetic reasons, suggested as one of the reasons in our survey, were not reported as a motivation for hymen reconstruction by the patients of the respondents.

#### Performing the surgery

As mentioned above, 80 respondents were ever requested to perform hymen reconstruction. Of this group, 42 gynaecologists (52%) stated they performed the surgery while 31 gynaecologists (39%) would not do it or would refer the patient, while seven (9%) would decide whether or not to do the surgery, depending on the story of the patient. Of the 29 respondents that were never confronted with the request, seven (24%) would perform it, eight (28%) would not perform the surgery, and 14 (48%) would refer the patient.

Of all those who (would) perform HR (49 respondents, 45%), 32 (65%) stated that they did it because it is the patient’s decision, independent of their own opinion. Twenty-two of them considered the risk situation of the patient a good reason to perform HR (45%). During the consultation, 65 respondents of those who did receive a request for HR (81%) did not discuss any alternatives that could possibility replace a surgery; while 15 (19%) indicated they suggested using alternatives such as the ‘finger prick’ or blood vial.

#### Not performing the surgery

Of all the 109 respondents, including those who did and did not receive a request for hymen reconstruction, 53 respondents (49%) indicated they would reject the request. Reasons commonly cited for rejecting the request included that the surgery is not medically indicated (42%), that they felt that they did not have the required technical skills (28%), the surgery is considered as assisting in deceiving the future husbands and families (19%), and 5 indicated they did not perform any surgery (9%).

#### Follow up and complications

A follow-up consultation was carried out by 29 of the 42 doctors (69%) that had performed hymen reconstruction. Thirteen of these (45%) indicated that only a minority of the patients came back for follow up. For 15 (more than half) of these doctors (52%), the follow-up consisted of checking the wound, six of them (21%) asked for psychological aspects such as how the patient experienced the wedding night. Of those who did not perform a follow-up consultation (48%), eight (40%) indicated that they thought it was unnecessary for such a non-invasive type of procedure and 3 (15%) said their patient did not want any follow up.

Complications while performing hymen reconstruction, were experienced by three of 42 doctors (7%) that performed the surgery. In one of these cases, a haemorrhage necessitated a new operation to achieve haemostasis. Other complications mentioned were dyspareunia and the formation of a haematoma.

## Discussion

Our survey among Flemish gynaecologists showed that the majority of our respondents have had requests to perform hymen reconstruction (73%). Knowledge and technical skills about hymen reconstructions were considered to be sufficient to perform the operation, by most of the respondents though HR did not seem to be integrated in a structured way in the medical curricula or training programme of most Flemish gynaecologists. Moreover, the majority of respondents did not see any need to introduce the subject of HR into the curricula, but would prefer to have guidance from the VVOG. Some insights were shared on the type of information needed, and refer to the psychosocial aspects of a patient’s request and the challenges in understanding the debates around HR. Respondents seemed to struggle with some issues related to hymen reconstruction, other than the clinical procedure. These include, being able to assess the risk for violence, if the surgery violates the bodily integrity of women and if the surgery can be considered as harmless and reversible.

It is remarkable to note that only few respondents (19%) discussed alternative options with the patient when hymen reconstruction is requested. Moreover, 55% of our respondents did not even discuss the anatomy and role of the hymen with the patient. However, counselling seems to influence the decision making process regarding HR. In some cases, it was found to discourage patients from requesting HR. A study done in the Netherlands among women who requested hymen reconstruction at a clinic (*n* = 82), found that extensive counselling played a role in dissuading 75% of the study participants from having the surgery done [7]. It would be interesting to explore further whether counselling should assist and if so, how counselling can assist in dissuading women from the operation in a Flemish context and whether and how capacity building on HR counselling should and can be introduced in training of health care professionals, likely to come across requests for HR, such as gynaecologists.

Although the majority of our respondents had requests for HR, it was striking to see that half of the respondents reject to perform HR. We could not assess from this survey where the women turn to when the surgery is refused. One of the main reasons cited for refusal was the fact that the procedure is considered ‘medically unnecessary’. This focus on the medical dimension is also found in the discussion on reimbursement of the HR surgery. The majority of our respondents were against reimbursement of the surgery. Many requests to abolish the reimbursement of hymen reconstructions have been introduced in the Belgian senate, for example in 2008 and 2010 [21, 25, 26]. Arguments put forward included that the principle of compulsory virginity prior to marriage is contrary to national ideas of basic rights or freedoms, that the surgery is medically not indicated and that this is not justifiable in view of the budgetary constraints of the social security system [27]. One of the authors of a proposal to abolish the reimbursement stated: “Why would we then refuse reimbursement for purely aesthetic operations, such as breast enlargements, and that are righteously considered as medically not necessary?” [21, 27]. The fact that a majority refused to perform HR, might indicate that the need for a HR for a number of women is not addressed, although this needs further investigation.

The above-mentioned arguments for abolishing reimbursement of HR in Belgium, seem to point to what has been described as “the risk of polarisation in a normative discussion” [4], whereby the medical perspective is dominant and moral or social dimensions of the practice are not taken into account. However, some of the very few evidence on views of women on HR indicate that there are a number of positive effects of HR, that go beyond the purely “medical” dimension of the issue [4].

The majority of respondents seemed to inquire about some socio-cultural aspects related to the patient’s request for the operation. However, only a minority believed that a patient is at risk of further violence, should her hymen not be intact at the time of marriage (47%). Research indicates that even though the procedure goes against the respondent’s personal principles; HCP might perform the surgery to prevent the patient from being harmed [9, 28]. It is difficult to ascertain why the majority of the respondents did not cite ‘fear of violence’ as a reason to perform hymen reconstruction surgery. It is possible that the respondents did not take these reports -  if they were made - seriously and relied more on their internal moral compass to decide if the HR was ‘medically necessary’ or not [29, 30]. It might also be the strong association between hymen reconstruction surgery and specific identities or ideologies of femininity, in particular in those ethnic communities where requests for HR come from in Belgium. These arguments are often cited when hymen reconstruction is framed as an act that supports patriarchy and women’s subordination [31–33]. Our survey indicates that a number of questions regarding the context of requests for HR by health professionals in Flanders need further attention.

Very little is known about the views of women requesting HR, their narratives on the consequences of a non-intact hymen and the positive effects of the surgery. Some notable examples of the empirical evidence stemming from the Netherlands and Tunisia [4, 6], are certainly lacking for Belgium/Flanders.

Seven percent of the respondents mentioned complications, following the surgery, which include haemorrhage, dyspareunia and formation of haematoma. The complications rates of the surgery are rarely discussed in the literature, and include bleeding and minor infections [4]. However, the true rate of complications is unknown, although the risks are assumed to be small [4]. This, again, needs further research that could assist in providing an evidence base to underpin the many debates around HR.

The majority of the respondents, who performed the operation, were able to perform a follow up consultation (29 of the 42 HCP). This is contrary to other studies that mentioned difficulties to accurately report the rate of complications, as most patients never return for ‘follow-up’ appointments [34]. However, from our survey we could not assess the reasons for our respondents being able to do follow up consultations.

## Strengths and weaknesses of the study

Although we reached with our survey nearly ¼ of the total population of the gynaecologists of the professional organisation VVOG, we are aware that no conclusions or inferences about causality or predictors of practices can be drawn from this modest survey. Given that 73% of our respondents have already been confronted with requests for HR, the findings of this survey cannot be generalised and we cannot exclude that the group of non-respondents might have other views. We also cannot exclude that there was a recall bias.

The primary purpose of the study was to provide insights on the knowledge, views/opinions and experiences of gynaecologists on hymen repairs. Given the lack of empirical evidence on how HCPs deal with hymen reconstructions, we nevertheless consider our study as valuable, as it provides some important insights in how gynaecologists confronted with HR are approaching such requests in Flanders. Our findings might contribute to the provision of evidence to underpin the debate among health care professionals on hymen repair.

## Conclusion

Our paper contributes to expanding the much-needed empirical evidence regarding health care professionals’ views on hymen reconstruction, by sharing insights of a survey among Flemish gynaecologists on this issue.

Our paper showed that many Flemish gynaecologists are likely to encounter requests for hymenoplasty, and suggests that a number of women are not receiving adequate responses to their requests to restore their hymen. There seems to be a lack of guidance and debate on all dimensions of HR (notably on social and moral dimensions). This paper provides mere first trends, and revealed a number of complexities when gynaecologists are addressing HR in Flanders. Perhaps providing more background and contextualising of HR, and how to respond to it, might assist health care professionals in deciding what option, surgery or not, is in the best interest of the patient. If guidance is to be developed, a need expressed by our respondents, it might consider take into account the ethical and social dimensions in such a guiding document.

The findings also show the need for qualitative research to explore and understand the complexities surrounding HR, that were raised by health care professionals’ responses in this study.

And finally, but most importantly, views and opinions of women on HR are notably lacking in Flanders. Qualitative research is urgently needed to document these women’s views. We consider such research as vital in providing input for any future development of guideline or policy on HR.

## Additional file

Additional file 1: Questionnaire in English. (PDF 71 kb)

## Abbreviations

CASP: Critical Appraisal Skills Programme
GGOLFGB: Gynécologues Obstétriciens de Langue Française de Belgique
HCP: Health Care Professionals
HR: Hymen reconstructions
