# Supplementary material for: ‘Doing hymen reconstruction’: an analysis of perceptions and experiences of Flemish gynaecologists
Source: BMC Womens Health. 2018 Jun 13;18:91. doi: 10.1186/s12905-018-0587-z (PMC6001123; doi:10.1186/s12905-018-0587-z)
Supplement: Supplementary file 1 — Questionnaire in English. (PDF 71 kb) [file 12905_2018_587_MOESM1_ESM.pdf]

1) What is your age?
    - 30 - 40 years;
    - 41 - 50 years;
    - 51 - 60 years;
    - 61 - 70 years

2) What is your gender?
    - Man
    - Woman

3) You are employed in:
    - Big city (Brussels, Antwerp or Ghent);
    - Medium-sized city;
    - Rural area;
    - Other

4) You work in a:
    - Private practice;
    - General Hospital,
    - University Hospital

5) Have you already had patients requesting hymen reconstructions?
    - Yes
    - No

6) How many requests for hymen repair did you receive in the last 12 months?
    - None,
    - 1,
    - 2,
    - 3,
    - 4,
    - 5,
    - 6-10,
    - 11-20,
    - More than 20

7) Do you request the following information from the patient:
    - Religion,
    - Ethnicity,
    - Age,
    - Relationship status (cohabiting, married, single),
    - Motivation for request for hymen reconstruction,
    - General knowledge of the patient about genitals and hymen,
    - Confidential person of the patient (someone who is close to the patient e.g. family, friend who can help her after surgery or where she go for advice)
    - Other

8) Do you ask for the reason why the women / girls want to undergo a hymen reconstruction?
   - Yes
   - No

9) Why do you ask (or not) for the reason why the women / girls want to undergo a hymen reconstruction?
   - ……………

10) What are the reasons that the women / girls indicate?
   - Religious prescription,
   - Cultural customs,
   - Pressure from the family,
   - For fear of violence,
   - Out of respect and affection for the family,
   - Out of respect for the future husband,
   - Cosmetic reasons,
   - Other

11) What do you do in case of a request for hymen reconstruction?
   - I do the surgery,
   - I do not perform the intervention,
   - I refer to others,
   - Other reason

12) Which items do you communicate with the patient?
   - I counsel the patient on the following alternatives: virginity ovule (A vaginal gelatin capsule that can simulate bleeding)
   - I counsel the patient on the following alternatives: Prick in finger (The woman pricks with a needle in the finger so that she can spread a few drops of blood on the sheet.)
   - I counsel the patient about other alternatives.
   - I inform the patient about my personal concerns but make it clear that she has the right to decide.
   - I am talking about the possible psychological consequences of the hymen reconstruction. (This means that the patient can develop feelings of guilt towards the partner due to unfairness of her virginity status)
   - Other

13) If you refuse or refer the patient, for what reason do you do that?
   - I do not have the necessary knowledge and technical skills to carry out such interventions.
   - I do not perform any surgical procedures.
   - The procedure is not medically indicated.
   - The intervention contributes to deception of the future marriage partner and the families involved.
   - Performing the intervention means maintaining the myth that every hymen will bleed at first coitus.

- The intervention contributes to the maintenance of a double standard. (That is, a virginity test for women and that it is not necessary for men.)
- The intervention violates the right to physical integrity and self-determination of women.
- Others

14) If you do the surgery, which is your motivation?
    - That it is the woman who has the right to decide on her own body, independent of your personal opinion.
    - That the woman is in danger, she can become a victim of honor killings.
    - That it is a small, reversible, innocent intervention without risks.
    - That the failure to operate maintains the double standard.
    - That it is an intervention like any other, I just execute them.
    - Others

15) Have you already experienced complications after carrying out such procedures?
    - Yes,
    - No,
    - Not applicable

16) If you have already experienced complications, what are the most common complications?
    - ……………………

17) Do you follow-up?
    - Yes,
    - No, go to 18
    - Not applicable

18) Why do you not do follow-up?
    - The patient is not open to this: she has made it clear that she would rather not take part in the follow-up.
    - Follow up is not necessary for this surgery
    - Other

19) Do you know the number of patients coming for a follow up consultation?
    - ……………..

20) If you do follow-up, what does it consists of?
    - ………………..

21) I register this procedure under:
    - Vulvo-vaginal plastic surgery
    - Surgery on the abdomen,
    - Not applicable
    - Other

22) If a patient would request a hymenreconstruction, I would:

- Perform the surgery,
- Not perform the surgery,
- Refer the patient,
- Other

23) If I were requested to do a hymen reconstruction, I would not carry out the procedure for the following reasons:

- I do not have the necessary knowledge and technical skills to carry out such interventions.
- I do not perform any surgical procedures.
- The procedure is not medically indicated.
- The intervention contributes to deception of the future marriage partner and the families involved.
- Performing the intervention means working on the myth that every hymen will bleeds at first coitus.
- The intervention contributes to the maintenance of a double standard. (That is, a virginity test for women and that it is not necessary for men.)
- The intervention violates the right to physical integrity and self-determination of women.
- Other

24) If I were requested to do a hymen reconstruction, I would perform the procedure for the following reasons:

- The woman has the right to decide on her own body, independent of my personal opinion.
- The woman is in danger, she can become a victim of honor killings.
- It is a small, reversible, innocent intervention without risks.
- The failure to operate maintains the double standard.
- It is an intervention like any other, I just execute it
- Other

25) Indicate the answer that is closest to your personal opinion:

	**Totally disagree**	**Disagree**	**Neutral**	**Agree**	**Totally agree**
I have the necessary knowledge and technical skills to carry out such operations					
The procedure is not medically indicated					
The intervention violates the right					

to physical integrity and self-determination of the woman					
The intervention means contributing to the myth that every hymen will bleed at first coitus					
The intervention contributes to the deception of the future marriage partner and the families involved					
The intervention helps to maintain a double standard. (i.e. a virginity test for women and that it is not necessary for men)					
The woman has the right to decide on her own body. My personal opinion about the operation is completely separate from this					
The woman is in danger, she can become a victim of honor killings					
It is a small, reversible, innocent intervention without risk					
Failure to operate					

maintains the double standard					
This procedure is an intervention like any other, I just execute them					
I think that hymen reconstructions maintain the virginity myth. (The virginity myth is the rumor that every girl should bleed at the first coitus)					

26) I think that such interventions should be reimbursed by the health insurance
   - Yes
   - No

27) If you have any comments about the previous question, you can mention them here
   …………………..

28) Was hymen reconstruction part of your basic training?
   - Yes
   - No

29) If you have any comments about the previous question, you can mention them here.
…………………..

30) Was hymenreconstruction part of your specialization training?
   - Yes
   - No

31) If you have any comments about the previous question, you can mention them here:
   ……………………

32) Would you rather have had more information about this procedure during your studies?
   - Yes
   - No

33) If you have any comments about the previous question, you can mention it here:
……………..

34) Do you find it useful to include this subject in the curriculum?
- o Yes
- o No

35) If you have any comments about the previous question, you can mention them here:
……………..

36) Are you familiar with any guidelines from Belgian or international organizations regarding hymen reconstructions?
- o Yes
- o No

37) If yes, which guideline was this?
……………

38) I think that the VVOG (Flemish Association of Gyn/obs) should draw up a guideline on hymenreconstructions.
- o Yes
- o No

39) If you have any comments about the previous question, you can mention them here:
…………………………..

40) If you would like to share any other comment, you can mention it here:
……………………..;
